# Hierarchically porous carbons as supports for fuel cell electrocatalysts with atomically dispersed Fe–N_*x*_ moieties†
†Electronic supplementary information (ESI) available. See DOI: 10.1039/c9sc01154d


**DOI:** 10.1039/c9sc01154d

**Published:** 2019-07-22

**Authors:** Lei Tong, Yu-Cheng Wang, Ming-Xi Chen, Zhi-Qing Chen, Qiang-Qiang Yan, Cheng-Long Yang, Zhi-You Zhou, Sheng-Qi Chu, Xinliang Feng, Hai-Wei Liang

**Affiliations:** a Hefei National Laboratory for Physical Sciences at the Microscale , Department of Chemistry , University of Science and Technology of China , Hefei , 230026 , China . Email: hwliang@ustc.edu.cn; b State Key Laboratory of Physical Chemistry of Solid Surfaces , Collaborative Innovation Center of Chemistry for Energy Materials , College of Chemistry and Chemical Engineering , Xiamen University , Xiamen , 361005 , China; c Institute of High Energy Physics , Chinese Academy of Sciences , Beijing 100049 , China; d Faculty of Chemistry and Food Chemistry , Center for Advancing Electronics Dresden , Technische Universität Dresden , 01062 Dresden , Germany . Email: xinliang.feng@tu-dresden.de

## Abstract

The development of high-performance non-platinum group metal (non-PGM) catalysts for the oxygen reduction reaction (ORR) is still of significance in promoting the commercialization of proton exchange membrane fuel cells (PEMFCs). In this work, a “hierarchically porous carbon (HPC)-supporting” approach was developed to synthesize highly ORR active Fe–phenanthroline (Fe–phen) derived Fe–N_*x*_–C catalysts. Compared to commercial carbon black supports, utilizing HPCs as carbon supports can not only prevent the formation of inactive iron nanoparticles during pyrolysis but also optimize the porous morphology of the catalysts, which eventually increases the amount of reactant-accessible and atomically dispersed Fe–N_*x*_ active sites. The prepared catalyst therefore exhibits a remarkable ORR activity in both half-cells (half-wave potential of 0.80 V in 0.5 M H_2_SO_4_) and H_2_–air PEMFCs (442 mA cm^–2^ at a working voltage of 0.6 V), making it among the best non-PGM catalysts for PEMFCs.

## Abstract 2

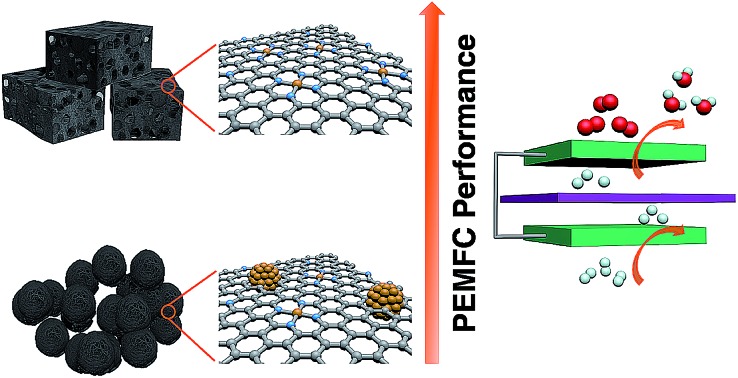
Fe–N_*x*_–C catalysts fabricated with hierarchically porous carbons instead of commercial carbon black demonstrate enhanced ORR performance under full-cell testing.

## Introduction

The high cost of Pt-based ORR catalysts has seriously hindered the commercialization of PEMFCs. In this regard, great efforts have been devoted to developing diverse non-PGM ORR catalysts with competitive performance.^1^,^2^ Among them, carbon-based materials with atomically dispersed Fe–N_*x*_ moieties (Fe–N_*x*_–C) are regarded as the most promising candidates to replace the state-of-the-art Pt/C catalysts,^3^–^7^ although so far Fe–N_*x*_–C catalysts are still much inferior to Pt/C, owing to the limited density of exposed Fe–N_*x*_ active sites and relatively poor transport properties of ORR-relevant species (H^+^, e^–^, O_2_, and H_2_O).^8^ It is therefore challenging but highly desirable to develop new approaches to prepare hierarchically porous Fe–N_*x*_–C catalysts for the efficient exposure of Fe–N_*x*_ sites and rapid transport of ORR species, which would considerably narrow the performance gap between non-PGM catalysts and Pt/C.

Fe–N coordination center-containing molecules supported on carbon materials (M_Fe–N_/C) are one of the most studied precursors for preparing Fe–N_*x*_–C catalysts. Pioneers of this field utilized conductive carbon black as a support and synthesized many outstanding Fe–N_*x*_–C catalysts.^9^–^14^ The high performance of these M_Fe–N_/C derived catalysts could be attributed to their porous structures with substantial mass transfer space and the high density of Fe–N_*x*_ sites. Nevertheless, post-treatments including acid leaching and a second heating process were normally required to maximize the amount of accessible active sites and accordingly improve the ORR activity. In addition, these approaches often failed to effectively control the evolution from molecular precursors to active Fe–N_*x*_ moieties and led to a heterogeneous environment with co-existing Fe-containing nanoparticles (Fe-NPs). These ORR-inactive inorganic nanoparticles were generated *via* the aggregation and growth of Fe atoms during pyrolysis, causing a low conversion ratio of precursors to active Fe–N_*x*_ moieties and the blockage of transport channels. An additional disadvantage of Fe-NPs is the potential harm to the long-term PEMFC operation *via* damaging the Nafion membrane by progressively dissolved ferrous cations.^15^,^16^ Recently, a silica shell-confined pyrolysis method was developed to increase the yield of Fe–N_*x*_ moieties during the thermal conversion of molecular precursors, though the tedious synthetic process was not able to completely prevent the formation of inactive nanoparticles.^17^,^18^ These challenges make it imperative to develop a facile and general strategy to prepare porous Fe–N_*x*_–C catalysts by minimizing Fe-NP formation and improving the mass transfer properties.

Herein, we report a facile and universal approach to prepare high-performance Fe–N_*x*_–C ORR catalysts for PEMFCs by using prefabricated hierarchically porous carbons (HPCs) as supports for loading the Fe–phen complex before pyrolysis treatment. The HPC supports are effective in avoiding the formation of Fe-NPs during the pyrolysis process. Simultaneously, the HPC-supported Fe–N_*x*_–C catalysts exhibit a hierarchically micro/meso/macro-porous structure with a high Brunauer–Emmett–Teller surface area (*S*_BET_) of 1723 m^2^ g^–1^, which is advantageous for exposing active sites and efficient mass transfer during PEMFC operation. These unique structural features lead to a high ORR activity with a half-wave potential (*E*_1/2_) of 0.80 V *versus* the reversible hydrogen electrode (RHE) in an acidic medium. Furthermore, the HPC-supported Fe–N_*x*_–C catalyst shows a high current density of 442 mA cm^–2^ at a working voltage of 0.6 V in H_2_–air PEMFCs.

## Results and discussion

**Fig. 1:**
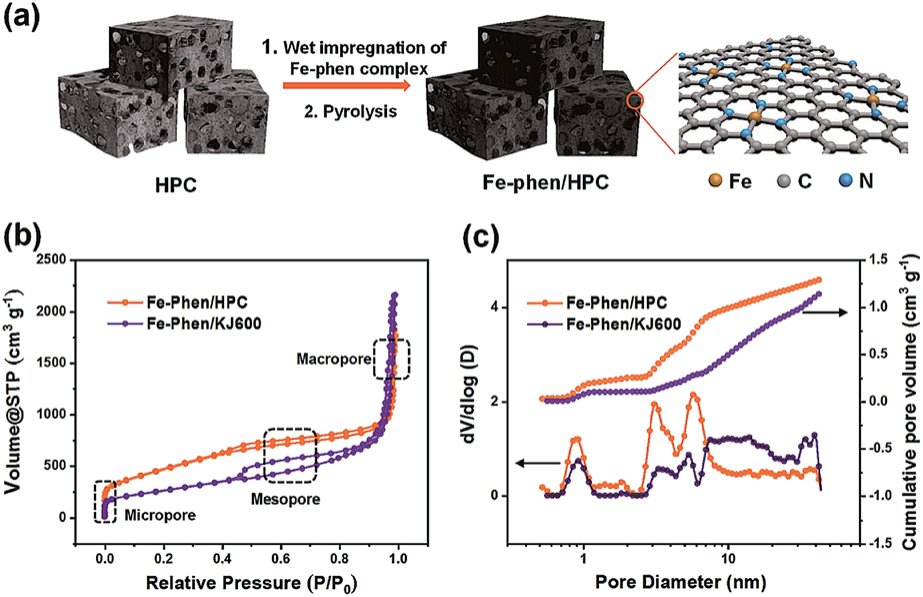
(a) Schematic illustration of the fabrication processes of Fe–phen/HPC. (b and c) Nitrogen adsorption/desorption isotherms (b) and pore size distributions and cumulative pore volumes (c) of Fe–phen/HPC and Fe–phen/KJ600.

The synthetic steps of the as-prepared catalysts include the preparation of HPC supports, wet impregnation of the Fe–phen complex into HPCs, and pyrolysis treatment of the composite at 800 °C in a nitrogen atmosphere (Fig. 1a). The HPC support was first prepared *via* pyrolyzing adenine under hypersaline conditions.^19^ N_2_ sorption isotherms revealed that the HPC support has a plot with a type IV pattern (Fig. S1†), including a rapid increase in the low-pressure region, a remarkable hysteresis loop, and a steep increase near *P*/*P*_0_ = 1, indicating the presence of micro-, meso-, and macro-pores, respectively.^20^ The specific surface area of the HPC support calculated by the Brunauer–Emmett–Teller (BET) method was 1966 m^2^ g^–1^. After impregnation of the Fe–phen complex and pyrolysis treatment at 800 °C, the *S*_BET_ of the prepared catalysts (referred to as Fe–phen/HPC) slightly decreased to 1723 m^2^ g^–1^. Importantly, the Fe–phen/HPC catalysts well inherited the hierarchically porous structure of the pre-fabricated supports (Fig. 1b). For comparison, commercial carbon black (Ketjenblack EC-600JD) was also used as a support to prepare Fe–N_*x*_–C catalysts (referred to as Fe–phen/KJ600) by the same process. The BET surface area of Fe–phen/KJ600 (949 m^2^ g^–1^) is much lower than that of Fe–phen/HPC, although Fe–phen/KJ600 also exhibited a hierarchically porous structure (Fig. 1b). Analysis of the pore size and volume showed that Fe–phen/HPC has a denser pore size distribution in the mesoporous range and a higher pore volume than Fe–phen/KJ600 (Fig. 1c).

**Fig. 2:**
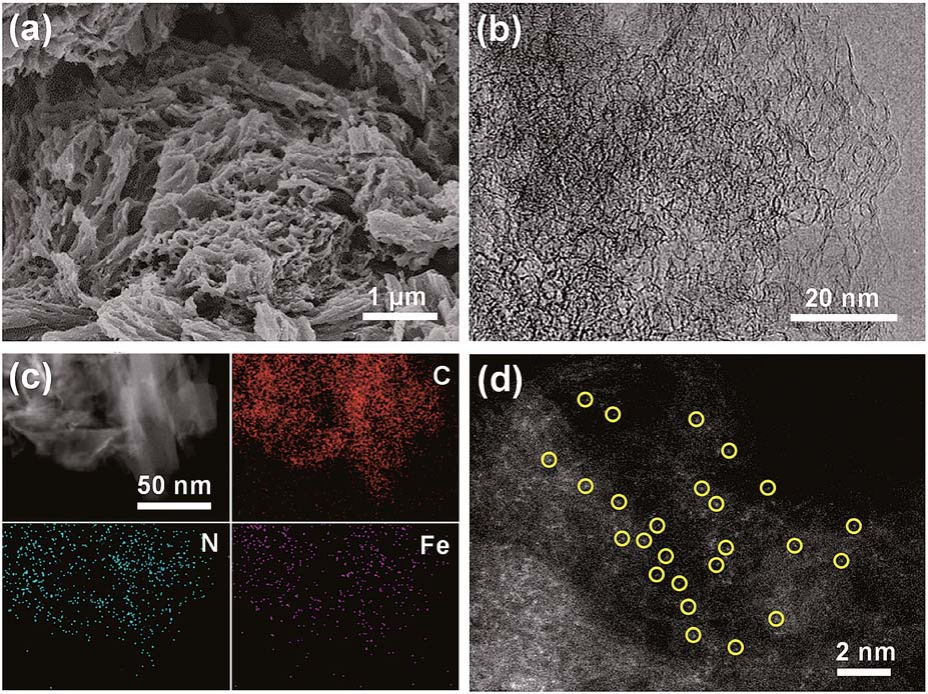
(a) SEM, (b) HRTEM, (c) STEM-EDS elemental mapping, and (d) aberration-corrected HAADF-STEM images of Fe–phen/HPC.

The scanning electron microscopy (SEM) image of Fe–phen/HPC showed bulk amorphous particles with micrometer sizes (Fig. 2a), whereas Fe–phen/KJ600 showed a similar morphology to the original KJ600 support with an agglomerated structure consisting of nanometer-sized carbon spheres (Fig. S2a†). Transmission electron microscopy (TEM) images verified the presence of widely distributed mesopores in both catalysts (Fig. 2b and b†). Energy-dispersive X-ray spectroscopy (EDS) analysis on a scanning transmission electron microscope (STEM) showed that both iron and nitrogen species were homogeneously distributed in Fe–phen/HPC without any inorganic nanoparticles (Fig. 2c). Meanwhile, the aberration-corrected high-angle annular dark-field STEM (HAADF-STEM) image revealed that Fe species existed exclusively in the form of atomically dispersed species in Fe–phen/HPC (Fig. 2d). In contrast, isolated Fe-NPs were observed for Fe–phen/KJ600 (Fig. S2c†). Both HPC and KJ600 derived catalysts were in the form of amorphous carbon, as indicated by X-ray diffraction (XRD) and Raman spectroscopy analyses (Fig. S3†).

Analysis of the X-ray photoelectron spectroscopy (XPS) N1s peak suggested the presence of pyridinic- (N1, 398.2 eV), pyrrolic- (N2, 399.75 eV), graphitic- (N3, 401 eV), and oxidized- (N4, 402.3 eV) nitrogen species in Fe–phen/HPC and Fe–phen/KJ600 (Fig. S4†). Among them, pyridinic and pyrrolic nitrogen species were proposed to be capable of coordinating with atomically dispersed Fe atoms to form active Fe–N_*x*_ sites.^21^,^22^ Iron contents of Fe–phen/HPC and Fe–phen/KJ600 determined by inductively coupled plasma atomic emission spectroscopy (ICP-AES) were 0.58 and 0.74 wt% respectively. Notably, ICP-AES analysis of Fe–phen/HPC after acid washing barely showed loss of Fe, whereas only 0.37 wt% Fe species in Fe–phen/KJ600 survived acid washing. It has been recognized that the Fe–N_*x*_ species embedded in the carbon matrix are stable under the acid environment, while the exposed Fe-NPs can be leached out by an acid solution.^15^

**Fig. 3:**
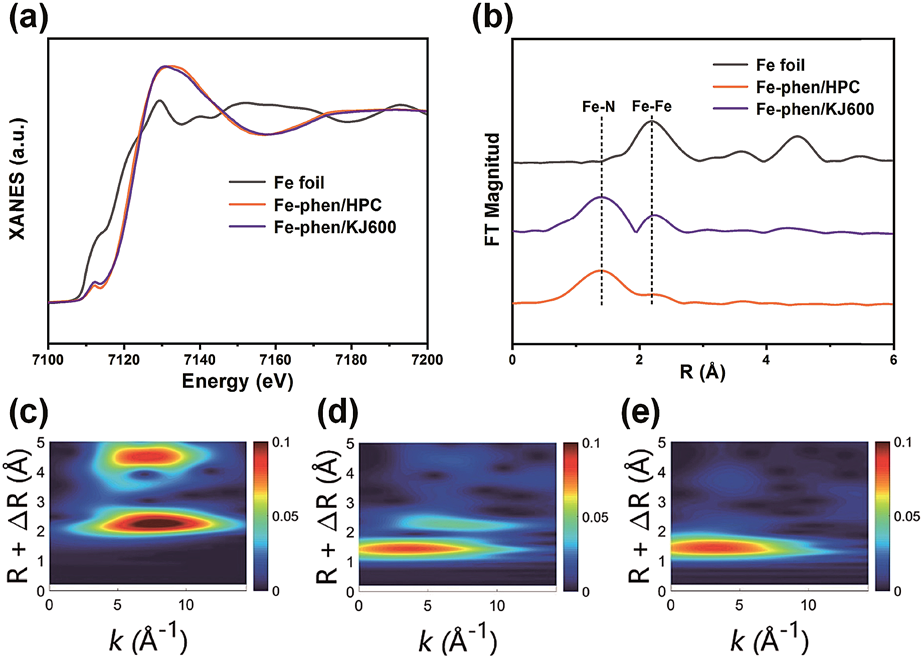
(a and b) Normalized Fe K-edge XANES spectra (a) and *k*^2^-weighted FT-EXAFS spectra (b) of Fe foil, Fe–phen/HPC, and Fe–phen/KJ600. (c–e) WT-EXAFS spectra of Fe foil (c), Fe–phen/KJ600 (d), and Fe–phen/HPC (e).

We then performed X-ray absorption near-edge structure (XANES) and extended X-ray absorption fine structure (EXAFS) measurements to further analyze the chemical environments of Fe species in the catalysts. The XANES spectra of Fe–phen/HPC and Fe–phen/KJ600 revealed a perceptible intensity at 7112 eV, which is associated to the 1s–4p_*z*_ transition feature for the square-planar structure with a *D*_4h_ symmetry,^23^ implying the presence of Fe–N_4_ sites in these two catalysts (Fig. 3a). The white line intensity of Fe–phen/HPC and Fe–phen/KJ600 was higher than that of Fe foil, indicating the dominance of ionic Fe species in the catalysts (Fig. 3a). The Fourier-transformed *k*^2^-weighted EXAFS (FT-EXAFS) analysis of Fe–phen/HPC demonstrated that there was only one apparent peak at 1.56 Å corresponding to the Fe–N contribution and no Fe–Fe characteristic peaks were observed (Fig. 3b), verifying the exclusive existence of atomically dispersed Fe–N sites on Fe–phen/HPC, whereas the FT-EXAFS analysis of Fe–phen/KJ600 showed both notable Fe–N and Fe–Fe peaks (Fig. 3b), further indicating the co-existence of Fe-NPs and Fe–N_*x*_ sites. Wavelet transform (WT) of EXAFS is a powerful tool to discriminate different atoms which may overlap substantially in the FT-EXAFS spectra.^24^ In good agreement with the FT analysis, the WT-EXAFS also verified the presence of metallic Fe species with a maximum feature at 8.0 Å^–1^ in Fe–phen/KJ600 and their absence in Fe–phen/HPC (Fig. 3c–e).

**Fig. 4:**
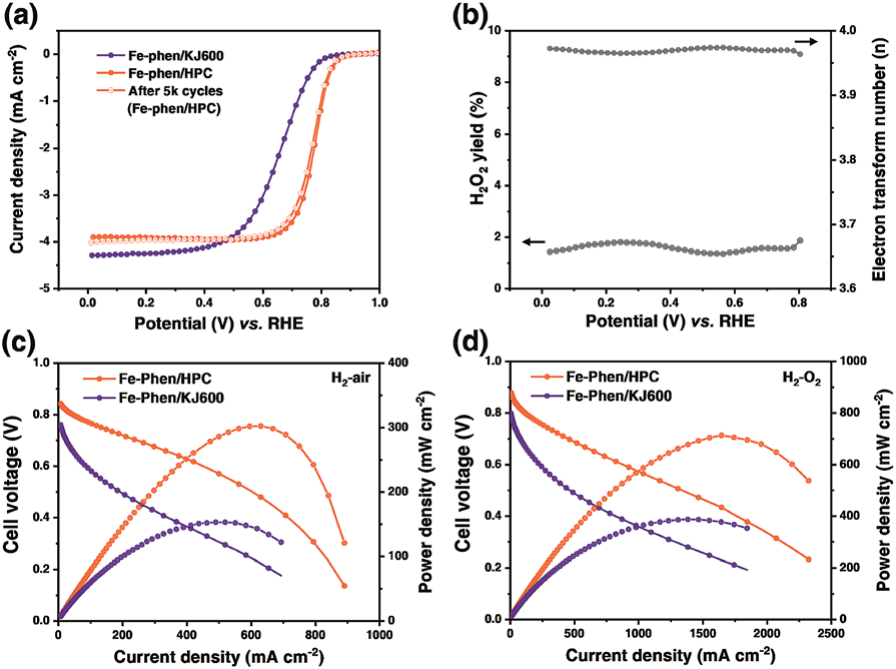
(a) Steady-state ORR polarization curves of Fe–phen/KJ600 and Fe–phen/HPC, and RDE stability of Fe–phen/HPC assessed after 5000 potential cycles. (b) H_2_O_2_ yield and calculated electron transfer number of Fe–phen/HPC. (c and d) Polarization and power density plots for the H_2_–air PEMFC (c) and H_2_–O_2_ PEMFC (d) with Fe–phen/HPC and Fe–phen/KJ600 as cathode catalysts.

The ORR performance of the catalysts was first evaluated using the rotating disk electrode (RDE) technique in 0.5 M H_2_SO_4_ (Fig. 4a). Fe–phen/HPC with a loading of 0.6 mg cm^–2^ displayed an (*E*_1/2_) of 0.78 V, which was significantly higher than that of Fe–phen/KJ600 (*E*_1/2_ = 0.66 V). Further increase of the Fe–phen/HPC catalyst loading to 0.8 mg cm^–2^ on the RDE resulted in an *E*_1/2_ of 0.8 V (Fig. S5†). The electrochemically active surface area (*S*_a_) of Fe–phen/HPC estimated from the double-layer capacitance (*C*_dl_)^11^,^25^ was 2.5 times higher than that of Fe–phen/KJ600 (Fig. S6†), indicating that Fe–phen/HPC could provide more accessible active sites during the ORR. In addition, the *E*_1/2_ loss of Fe–phen/HPC was only 7 mV after 5000 RDE potential cycles (Fig. 4a), indicating the high durability of the Fe–phen/HPC catalyst. Rotating ring-disk electrode (RRDE) testing revealed that Fe–phen/HPC maintained <2% yield of H_2_O_2_ at all potentials, corresponding to the high electron-transfer number of >3.95 (Fig. 4b). Apart from the superior ORR activity in acidic media, the Fe–phen/HPC catalyst also performed well in alkaline media, as confirmed by an *E*_1/2_ of 0.89 V in 0.1 M O_2_-saturated KOH solution in the RDE test (Fig. S7†).

RDE testing preliminarily revealed that the ORR activity of Fe–phen/HPC ranks among that of the best-performing non-PGM ORR catalysts.^5^,^10^,^11^,^26^–^34^ However, the high ORR activity obtained from the half-cell measurement cannot be directly associated with high PEMFC performance because of the different working conditions and environment.^5^ H_2_–air PEMFC testing was therefore carried out to evaluate the potential of the HPC derived catalysts for practical fuel cell applications. Fig. 4c shows the single cell polarization curves measured under an absolute H_2_–air pressure of 1.5 bar and a cathode loading of 2 mg cm^–2^. At an operating voltage of 0.6 V, Fe–phen/HPC exhibited an increase in the current density (442 mA cm^–2^) compared with Fe–phen/KJ600 (88 mA cm^–2^). Meanwhile, the peak power density of Fe–phen/HPC-Ad (301 mW cm^–2^) also outperformed that of Fe–phen/KJ600 (152 mW cm^–2^). To minimize mass transfer effects and better reveal the real fuel cell activity of the as-prepared catalysts,^5^ testing under an absolute H_2_–O_2_ pressure of 1 bar was also performed. As expected a much-improved performance could be achieved under H_2_–O_2_ conditions (Fig. 4d and Table S1†), the peak power density and current density at 0.6 V for Fe–phen/HPC reached 712 mW cm^–2^ and 875 mA cm^–2^ respectively.

We believe that the better polarization performance of Fe–phen/HPC in PEMFCs than Fe–phen/KJ600 is associated to not only the improved intrinsic ORR activity, but also the enhanced mass transport in the Fe–phen/HPC catalyst layer. To confirm this point, we plotted the concentration overpotential (*η*_C_) *vs.* current density curves determined by separating the cell voltage, which was given by Tafel's equation from the *iR*-corrected voltage.^35^ As expected, the Fe–phen/HPC cathode exhibited better mass-transport properties than the Fe–phen/KJ600 cathode as revealed by the lower *η*_C_ of Fe–phen/KJ600 than that of the Fe–phen/HPC cathode (Fig. S8†).

Apart from activity, the long-term durability is another critical property for Fe–N_*x*_–C catalysts. Unfortunately, similar to most reported Fe–N_*x*_–C catalysts,^15^,^36^,^37^ the Fe–phen/HPC catalyst also displayed an obvious performance loss in the first 60 h under H_2_–air operating conditions (Fig. S9†). In short, the activity of the HPC derived cathode in PEMFCs can be compared favorably to that of most recently reported non-PGM catalysts (Tables S2 and S3†), but challenges still remain to achieve acceptable PEMFC stability.

To further demonstrate the wide applicability of our strategy for preparing highly active Fe–N_*x*_–C catalysts, another HPC (denoted as HPC-Dcb) with a high *S*_BET_ of 1803 m^2^ g^–1^ was prepared^38^ and used as a support to prepare the Fe–N_*x*_–C catalyst (denoted as Fe–phen/HPC-Dcb). The *S*_BET_ and *C*_dl_ of Fe–phen/HPC-Dcb were 1413 m^2^ g^–1^ and 288 mF cm^–2^, respectively. The amorphous carbon structure and hierarchical micro-/meso-porous structures of Fe–phen/HPC-Dcb were confirmed by XRD, Raman spectroscopy, N_2_ adsorption/desorption isotherms, SEM, and HRTEM analyses (Fig. S3 and S10†). Individually dispersed Fe atoms in the catalyst were observed by STEM measurements (Fig. S11†). XANES and FT- and WT-EXAFS spectra demonstrated the exclusive presence of atomically dispersed Fe–N_*x*_ sites in Fe–phen/HPC-Dcb (Fig. S12†). The Fe–phen/HPC-Dcb catalyst also displayed remarkable ORR activity under half-cell conditions (Fig. S13a and b†), including a high *E*_1/2_ of 0.78 V, a low H_2_O_2_ yield of below 2% at all potentials, and outstanding durability (only 10 mV decrease of *E*_1/2_ after 5000 RDE potential cycles). Moreover, H_2_–air PEMFC tests showed that Fe–phen/HPC-Dcb possessed a higher fuel cell cathode activity than Fe–phen/KJ600 (Fig. S13c and Table S1†). Note that the Fe–phen/HPC-Dcb cathode displayed a lower current density in the mass-transfer controlled region compared to Fe–phen/HPC (Fig. S13d†), probably due to the lack of macropores in Fe–phen/HPC-Dcb, which limited the mass transfer efficiency during the ORR.^5^,^39^

## Conclusions

In summary, we report an efficient strategy to boost the ORR activity of Fe–N_*x*_–C catalysts by utilizing HPCs as the supports. Compared to the catalyst fabricated with traditional carbon black supports, the HPC supported Fe–N_*x*_–C catalyst exhibited remarkably enhanced ORR performance, as revealed by both RDE and PEMFC measurements. Such a significant improvement of the ORR performance is associated with the increase of reactant-accessible active sites, which was achieved by utilizing HPCs as the carbon support to increase the formation of Fe–N_*x*_ sites and favor a porous morphology. We believe that the HPC-supporting strategy could be broadly effective to prepare other carbon-based catalysts with atomically dispersed metal sites for catalyzing a wide range of reactions.

## Conflicts of interest

There are no conflicts to declare.

## Supplementary Material

Supplementary information
