# Supplementary material for: Hierarchically porous carbons as supports for fuel cell electrocatalysts with atomically dispersed Fe–Nx moieties
Source: Chem Sci. 2019 Jul 22;10(35):8236–40. doi: 10.1039/c9sc01154d (PMC6788509; doi:10.1039/c9sc01154d)
Supplement: Supplementary file 1 [file SC-010-C9SC01154D-s001.pdf]

Electronic Supplementary Material (ESI) for Chemical Science.
This journal is © The Royal Society of Chemistry 2019

Electronic Supplementary Information for

Hierarchically Porous Carbons as Supports for Fuel Cell Electrocatalysts with Atomically-Dispersed Fe-N_x Moieties

Lei Tong,[a‡] Yu-Cheng Wang,[b‡] Ming-Xi Chen,[a] Zhi-Qing Chen,[a] Qiang-Qiang Yan,[a] Cheng-Long Yang,[a] Zhi-You Zhou,[b] Sheng-Qi Chu,[c] Xinliang Feng[*d] and Hai-Wei Liang[*a]

[a] Hefei National Laboratory for Physical Sciences at the Microscale, Department of Chemistry, University of Science and Technology of China, Hefei, 230026, China

[b] State Key Laboratory of Physical Chemistry of Solid Surfaces, Collaborative innovation center of Chemistry for Energy Materials, College of Chemistry and Chemical Engineering, Xiamen University, Xiamen, 361005, China

[c] Institute of High Energy Physics, Chinese Academy of Sciences, Beijing 100049, China

[d] Department of Chemistry and Food Chemistry & Center for Advancing Electronics Dresden, Technische Universität Dresden, 01062 Dresden, Germany

Experimental Section

Synthesis of HPC supports. For the synthesis of HPC,[1] adenine (0.80 g) and $MgCl_2 6H_2O$ (8.0 g) were ground together in a glovebox. Then, the mixture was transferred into a quartz crucible and heated at 900 °C for 1h under the N_2 environment. After cooling to room temperature, the obtained product was washed with 1 M HCl and deionized water for three times, then dried in air at 80 °C overnight. For the synthesis of HPC-Dcb,[2] 1,4-dicyanobenzene (200 mg) and anhydrous $ZnCl_2$ (1.06 g) were mixed and transferred into quartz tube in a glovebox. Then the tube was evacuated, sealed and heated at 700 °C for 20 hours. The obtained product was washed and dried by the same process as above.

Synthesis of the Fe-N_x-C catalysts. Fe (II) acetate (5 mg) and 1, 10-phenanthroline monohydrate (100 mg) were first dissolved in 50 mL ethanol to form Fe-Phen complex at room temperature. Then, the as-prepared HPC support (100 mg) was added and the mixture was heated to 60 °C for 24h before the removal of ethanol by a rotary evaporator. The solid mixed powder was further dried in air at 80 °C overnight. After that, the dried powder was ground thoroughly and transferred into a ceramic crucible. Then, the Fe-Phen/HPC catalyst was obtained by heating the mixture at 800 °C for 2h

under N_2 atmosphere. Fe-Phen/HPC-Dcb and Fe-Phen/KJ600 were prepared by the same processes with HPC-Dcb and KJ600 as supports, respectively.

RDE and RRDE measurements. The half-cell ORR performance of the catalysts was determined by using a CHI Electrochemical Station (Model 760E) in the RDE and RRDE mode with a three electrode electrochemical cell. Ag/AgCl (3M KCl) and graphitic electrodes were chosen as reference and counter electrodes, respectively. The hydrogen redox test was performed in each RDE or RRDE measurement to complete the reversible hydrogen electrode (RHE) calibration. The ink of our prepared catalysts was obtained by sonicating the mixture consisting of 6 mg catalyst, 68 µL (5 wt. %) Nafion in 600 mL ethanol, and 355 mL deionized water for 1 hour. Then, the ink was drop-coated onto the working electrode (RDE: a glassy carbon disk with diameter 5.0mm; RRDE: a Pt ring with inner diameter of 6.25mm and outer diameter of 7.92mm, and a glassy carbon disk with diameter 5.61mm) with a loading of 0.6 mg cm^{-2} and dried at room temperature in air. After drying, the catalyst was activated by a cyclic voltammetry method (scan rates of 100 mVs^{-1} and potential ranges of 0.0-1.0 V vs. RHE) until a reproducible curve was obtained. Then, the catalyst was characterized by linear stair case voltammetry (LSCV) method with a potential step of 20 mV and a wait-periods of 25 s, scanning range from 1.0 to 0.0 V *vs.* RHE. All RDE and RRDE tests were conducted in 0.5 M H_2SO_4 at a rotation rate of 900 rpm. The ADT test was performed by cycling RDE from 0.6 to 1.0 V *vs.* RHE at a scan rate of 50 mV s^{-1}. The ring electrode potential of the RRDE was set to 1.3 V *vs.* RHE. Double layer capacitance (C_{dl}) values of the catalysts were obtained by cyclic voltammograms (CVs) in the non-faradic potential range of 0.975 to 1.025 V *vs.* RHE with scan rates from 1 to 5 mV s^{-1}, the current density obtained from the upper branch of CVs at 1.0 V *vs.* RHE was plotted against the scan rate, and the slope of the curve was the value of C_{dl}.[3] The mass related kinetic current density (J_K) of catalyst at 0.8 V *vs.* RHE was calculated according to: $J_k = J \times J_L / ((J_L - J) \times L_{catalyst})$. J, J_L, and $L_{catalyst}$ represent the current density at 0.8 V *vs.* RHE, the diffusion limited current density, and the catalyst loading respectively.

PEMFC measurements. The ink with 3:1 mass ratio of Nafion/catalysts was sprayed

on a carbon paper (Toray 060) with a catalyst loading of 2 mg cm^{-2}. After dried, this carbon layer was used as the cathode directly. Similarly, the anode was prepared by coating 40 wt% Pt/C (Johson Mattthey) with a loading of 0.4 mg_{Pt} cm^{-2}. Then, the anode, cathode, and NRE211 Nafion membrane were hot pressed together to fabricate the membrane electrode assembly (MEA) with an active area of 2.25 cm^2. Polarization curves were obtained by the Model 850e fuel cell test system (Scribner Associates Inc.) under a 100% relative humidity environment. The test temperature and the flow rate of each gas were controlled at 80 °C and 0.2 slpm, respectively. The bar pressures of anode and cathode under the H_2-O_2 condition were both set at 1 bar. For the H_2-air condition, their back pressures were improved to 1.5 bar.

Characterization. XRD was performed with Japan Rigaku DMax-γA rotation anode x-ray diffractometer using Cu K-α radiation. N_2 sorption analysis was conducted on a Quantachrome Autosorb-iQ instrument, the quenched solid density functional theory (QSDFT) method was applied to reveal the pore size and volume distributions of the catalysts in the micropore and mesopore ranges. XPS was performed on a Thermo Scientific ESCALAB (ESCALab MKII) with an excitation source of Mg Ka radiation (1253.6 eV). Low-magnification HAADF-STEM, HRTEM, and elemental mapping images were obtained by a JEM-2100F instrument with an acceleration voltage of 200 kV. Atomic resolution STEM images were obtained using JEM ARM200F (S) operated at 200 kV. The ICP-AES measurement was carried out using Optima 7300 DV. X-ray absorption fine structure data of Fe K-edge were acquired at 1W1B beamline of the Beijing Synchrotron Radiation Facility operated at 2.5 GeV and 200 mA and on the BL14W1 beam line of Shanghai Synchrotron Radiation Facility (SSRF) operated at 3.5 GeV and 220 mA. The raw data analyses were conducted by using the Athena program in IFEFFIT software package. The energy was first calibrated, then the pre-edge background of the spectrum was subtracted and post-edge was normalized. k^2-weighted EXAFS oscillations ranging from 1.8-9.5 Å were Fourier transformed to obtain a radial distribution function.

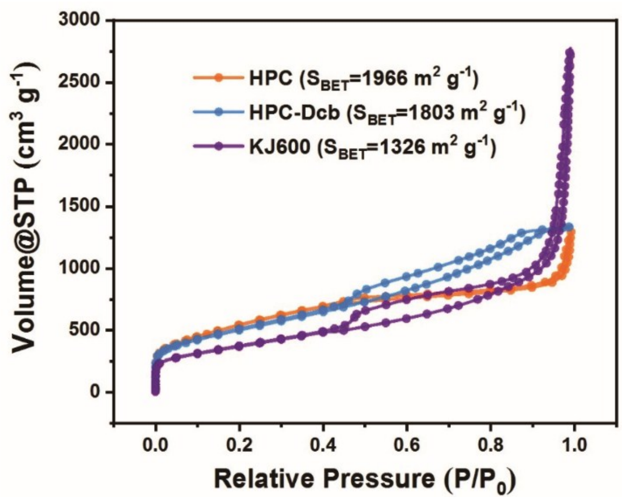

Figure S1. Nitrogen adsorption/desorption isotherms and calculated S_{BET} of HPC, HPC-Dcb, and KJ600.

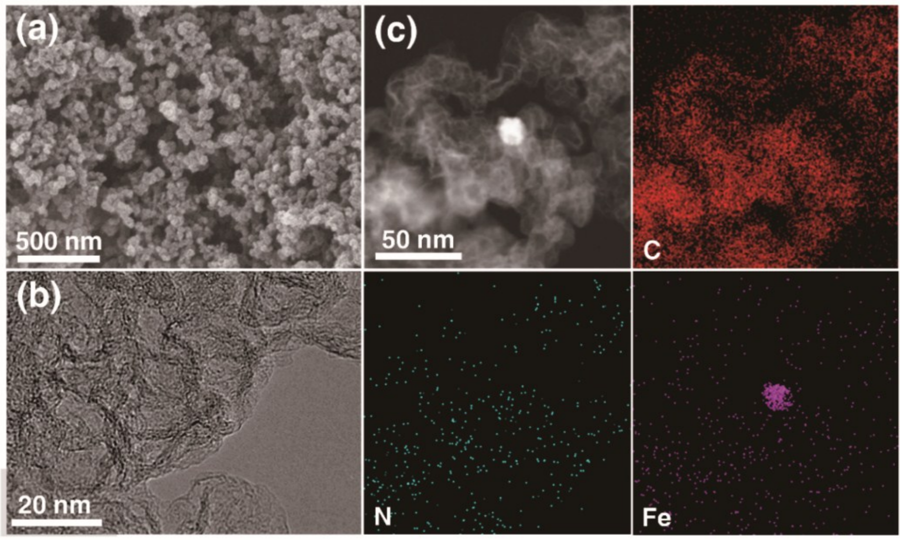

Figure S2. (a) SEM image, (b) HRTEM image, and (c) STEM-EDS elemental mapping image of Fe-phen/KJ600.

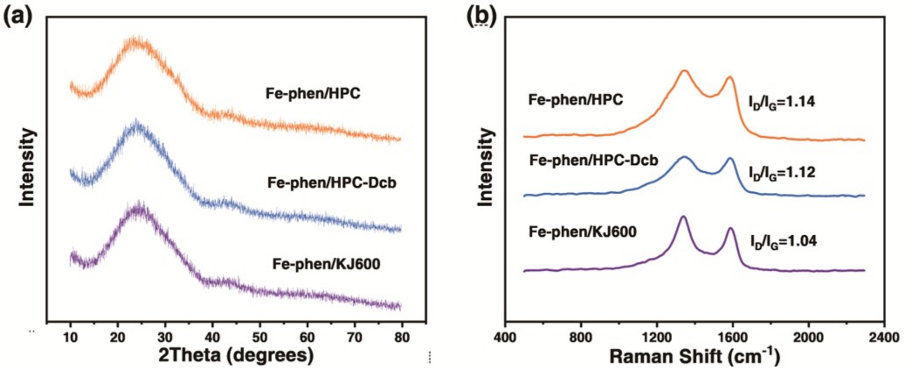

Figure S3. (a) XRD patterns and (b) Raman spectra of the as-prepared catalysts. The two broad peaks at 25° and 44° in XRD patterns are corresponding to the (002) and (101) reflections of amorphous carbon. For the Raman spectra, we observed the D and G bands positioned at 1,345 cm^{-1} and 1,595 cm^{-1}, which represent the hexagonally bonded carbon atoms inside the graphitic networks and the distorted carbon frames on the defect sites, respectively. Both catalysts displayed a stronger integral intensity of D-band than that of G-band, further suggesting that amorphous carbon is dominant in their structure.

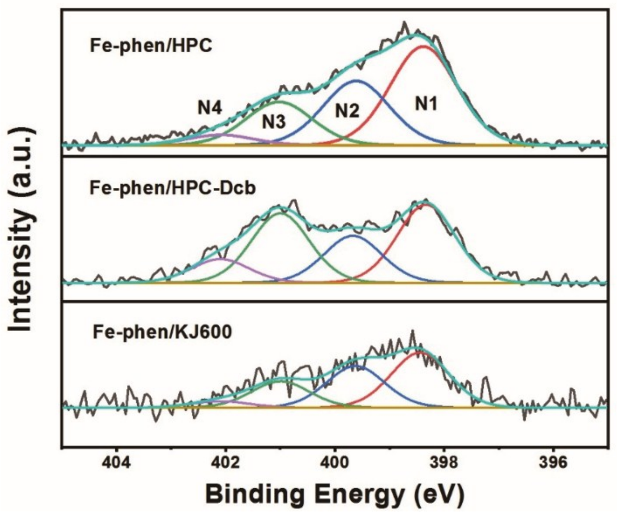

Figure S4. High-resolution XPS N1s spectra of the as-prepared catalysts.

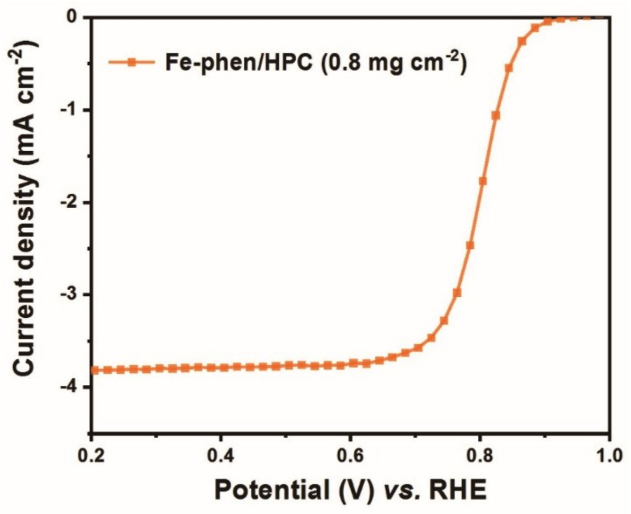

Figure S5. Steady-state ORR polarization curve of Fe-phen/HCP in 0.5M H_2SO4.

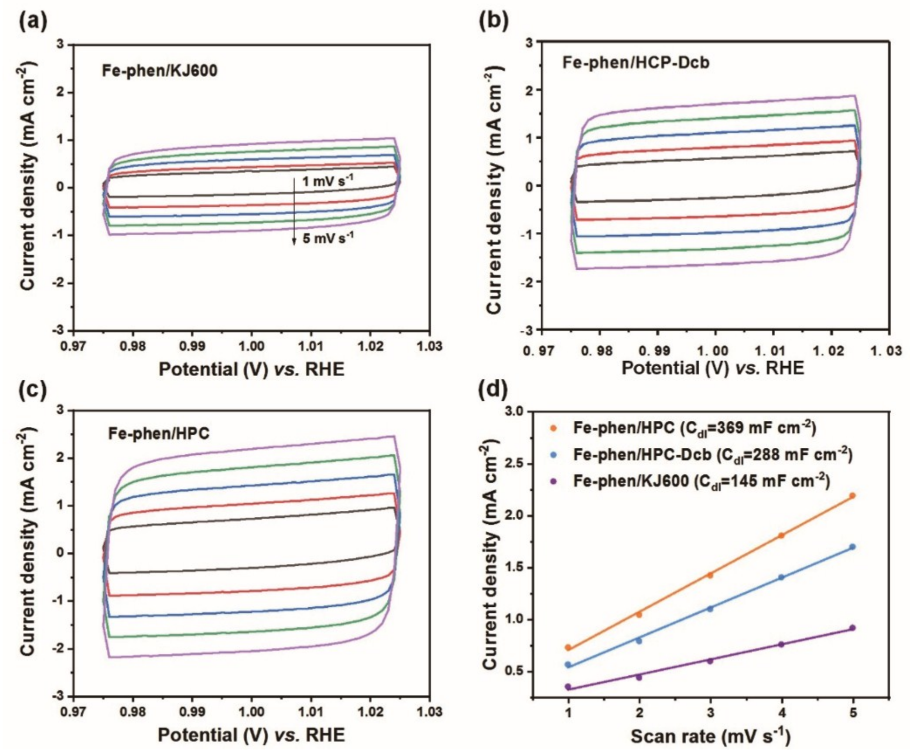

Figure S6. (a-c) Cyclic voltammetry (CV) plots of Fe-phen/KJ600 (a), Fe-phen/HCP-Dcb (b), and Fe-phen/HCP (c) at different scaning rates. (d) Estimated C_{dl} values calculated by linear fitting plots of current densities as a function of scan rates at a non-faradic potential (1.0 V vs. RHE).

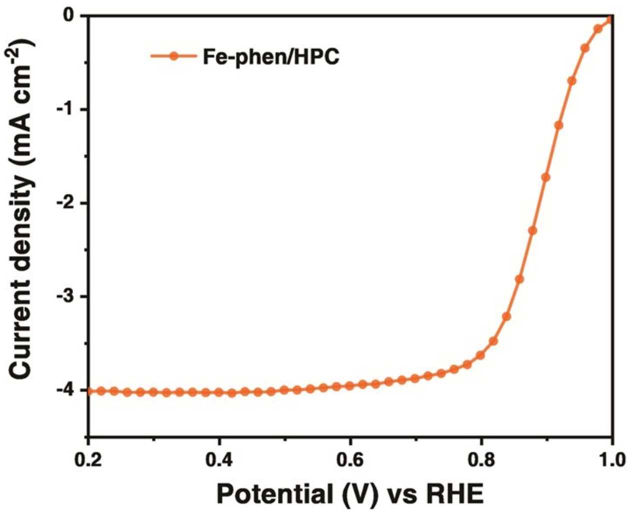

Figure S7. Steady-state ORR polarization curve of Fe-phen/HCP with a loading of 0.8 mg cm^{-2} in 0.1M KOH.

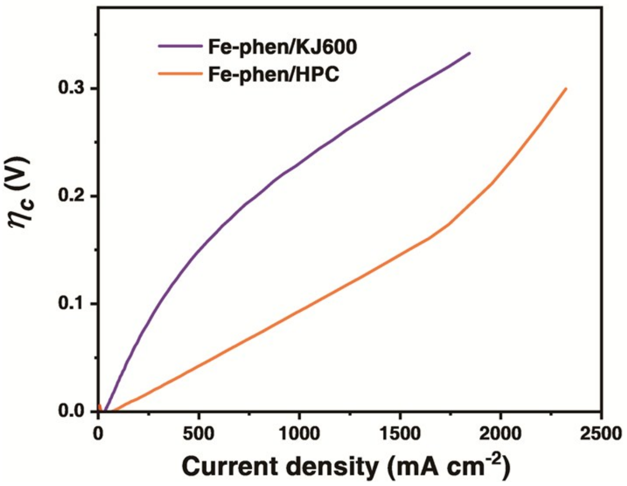

Figure S8. Concentration overpotential (η_C) vs. current density curves for the as-prepared catalysts.

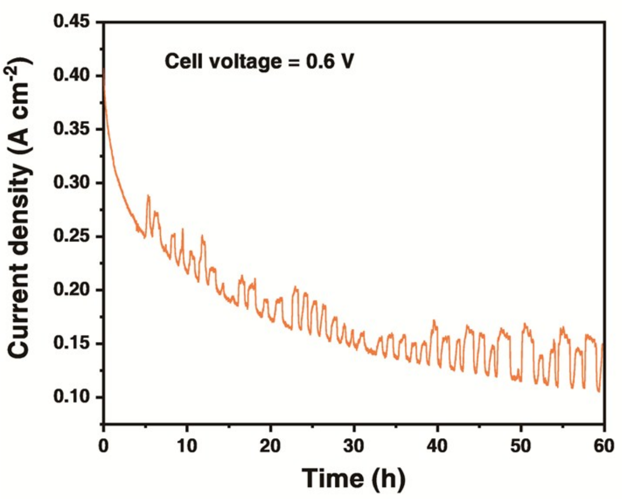

Figure S9. Stability test of for H_2-air PEMFC with Fe-phen/HPC as the cathode catalyst.

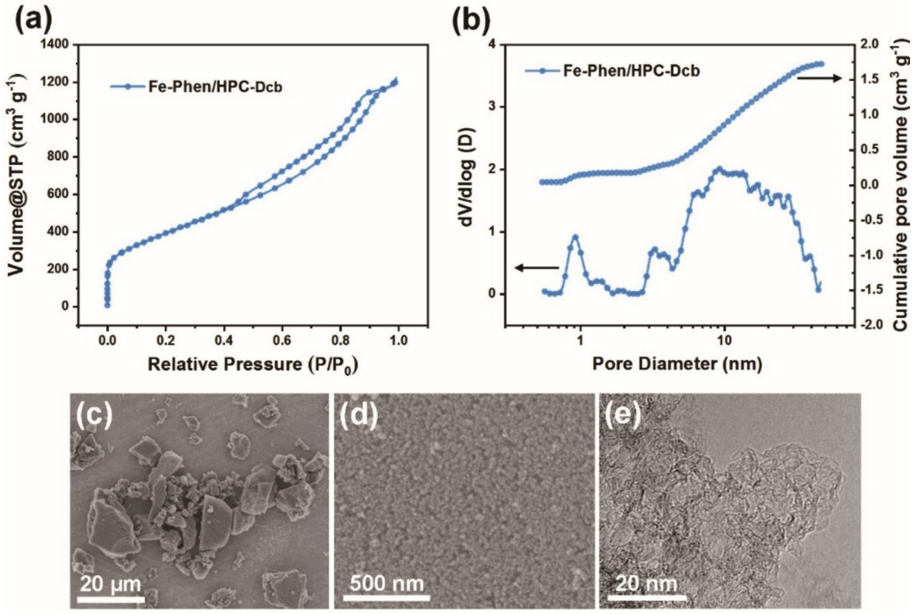

Figure S10. (a) Nitrogen adsorption/desorption isotherm, (b) pore size distribution and cumulative pore volume, (c, d) SEM images, and (e) HRTEM image of Fe-phen/HPC-Dcb.

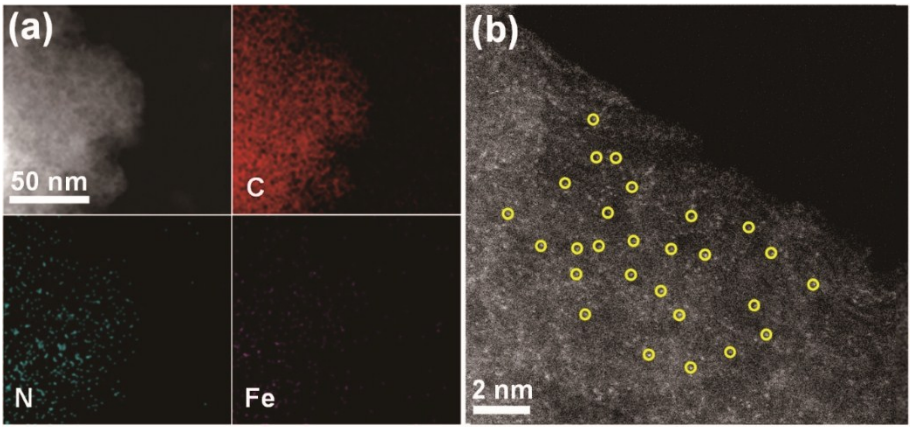

Figure S11. (a) STEM-EDS elemental mapping images and (b) aberration corrected HAADF-STEM image of Fe-phen/HCP-Dcb.

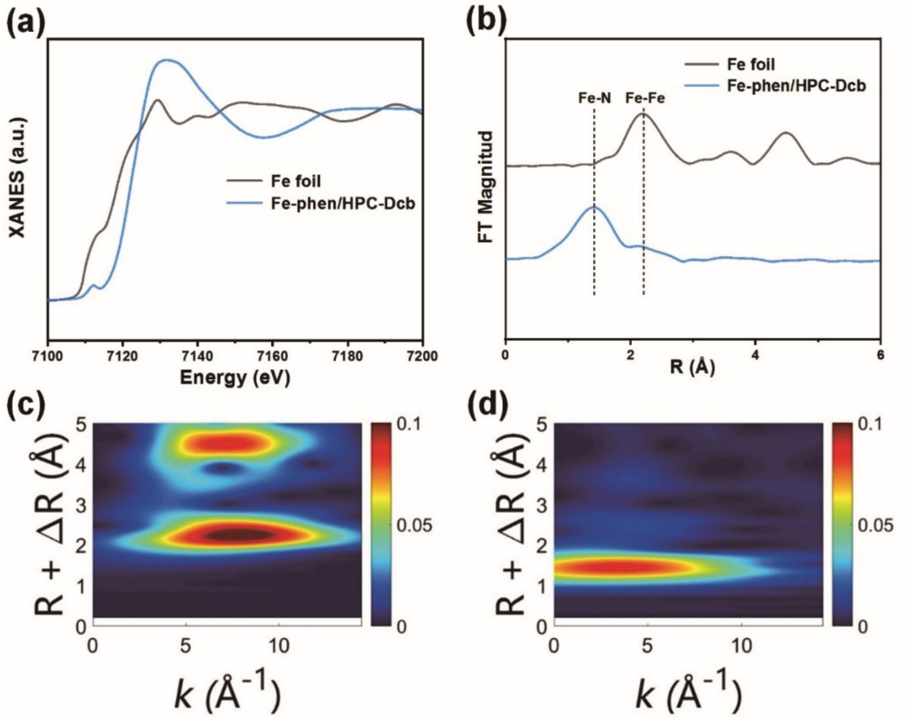

Figure S12. (a) Normalized Fe K-edge XANES spectra and (b) k^2-weighted FT-EXAFS spectra of Fe foil and Fe-phen/HPC-Dcb. (c, d) WT-EXAFS spectra of Fe foil and Fe-phen/ HCP-Dcb, respectively.

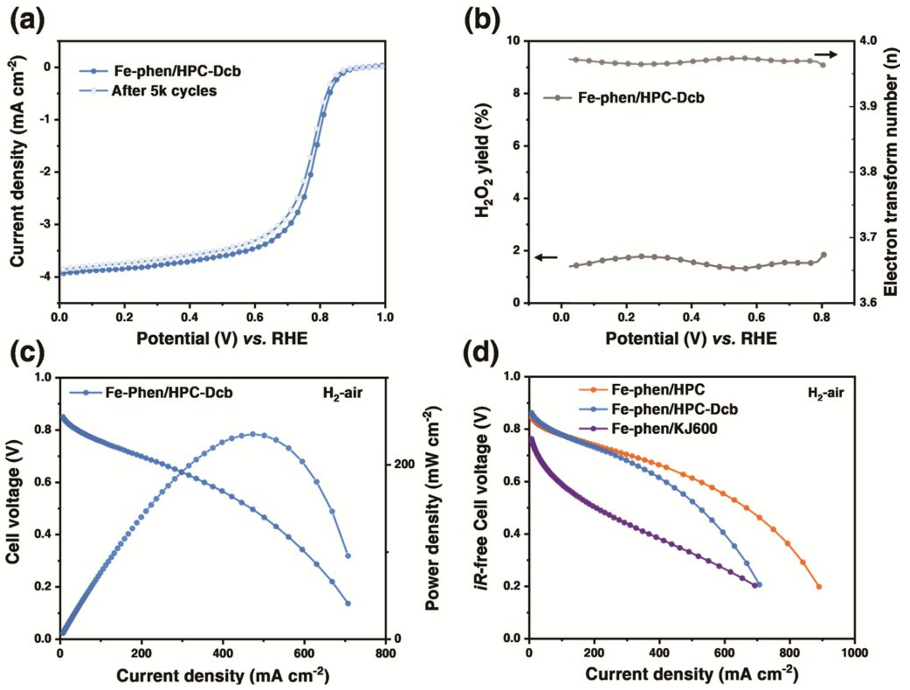

Figure S13. (a) Steady-state ORR polarization curves of Fe-phen/HCP-Dcb before and after 5 000 RDE potential cycles. (b) H_2O_2 yield and calculated electron transfer number of Fe-phen/HCP-Dcb. (c) Polarization and power density plots for H_2-air PEMFC with Fe-phen/HPC-Dcb as the cathode catalyst. (d) *iR*-free polarization plots for H_2-air PEMFC with Fe-phen/HPC, Fe-phen/HPC-Dcb, and Fe-phen/KJ600 as cathode catalysts.

Table S1. Performance of H_2-air and H_2-O_2 PEMFCs with the prepared materials as cathode catalysts.

Catalysts	Current density@0.6 V (mA cm^{-2})		Peak power density (mW cm^{-2})	
	H_2-air	H_2-O_2	H_2-air	H_2-O_2
**Fe-phen/HPC**	442	875	301	712
**Fe-phen/HPC-Dcd**	355	\	233	\
**Fe-phen/KJ600**	88	219	152	384

Table S2. Performance comparison of H_2-air PEMFCs employing Fe-N_x-C materials as the cathode catalysts under 353 K.

Catalysts	Loading (mg cm^{-2})	Back pressure (bar)	Current Density @ 0.6 V (mA cm^{-2})	Peak power density (mW cm^{-2})	Ref
**Fe-phen/HPC**	**2**	**1.5**	**442**	**301**	**This work**
**Fe-N-C-Phen-PANI**	4	1.37	611*	380	[3]
**(CM+PANI)-Fe-C**	4	1	645*	420	[4]
**FePhenMOF-ArNH$_3$**	2	2	600*	400	[5]
**Fe/TPTZ/ZIF-8**	1.14	1	500	300*	[6]
**Fe-PANI/ C-Mela**	4	2.06	408*	330	[7]
**Fe/oPD–Mela**	3	2.06	300*	270	[8]
**Fe/PI-1000-III-NH$_3$**	4	2	430*	320	[9]

*These values were not directly given in the papers and thus obtained by digging the data from the polarization curves.

Table S3. Performance comparison of H_2-O_2 PEMFCs employing Fe-N_x-C materials as the cathode catalysts under 353 K.

Catalysts	Loading (mg cm^{-2})	Back pressure (bar)	Current density @ 0.6 V (mA cm^{-2})	Peak power density (mW cm^{-2})	Ref
**Fe-phen/HPC**	**2**	**1**	**875**	**712**	**This work**
**Fe-N-C-Phen-PANI**	4	1.37	1433	1060	[3]
**(CM+PANI)-Fe-C**	4	2	1038*	940	[4]
**Fe/TPTZ/ZIF-8**	1.14	1	1050	750*	[6]
**CNT/PC**	3	1	550	580	[10]
**PFeTTPP-1000**	4.1	1.5	601*	730	[11]
**CoFe-N-C**	4	2.8	390*	420	[12]
**ZIF-FA-CNT-p**	4	1	880	820	[13]
**Fe2-Z8-C**	2.8	2	1600	1110	[14]
**Fe/N/C-SCN**	4	2	1410*	1030	[15]
**SA-Fe/NG**	2	2.5	850	823	[16]

*These values were not directly given in the papers and thus obtained by digging the data from the polarization curves.

References

1. J. Pampel, A. Mehmood, M. Antonietti and T. P. Fellinger, *Mater. Horizons*, 2017, **4**, 493-501.
2. P. Kuhn, A. Thomas and M. Antonietti, *Macromolecules*, 2009, **42**, 319-326.
3. X. Fu, P. Zamani, J.-Y. Choi, F. M. Hassan, G. Jiang, D. C. Higgins, Y. Zhang, M. A. Hoque and Z. Chen, *Adv. Mater.*, 2017, **29**, 1604456.
4. H. T. Chung, D. A. Cullen, D. Higgins, B. T. Sneed, E. F. Holby, K. L. More and P. Zelenay, *Science*, 2017, **357**, 479-484.
5. J. Li, S. Ghoshal, W. Liang, M.-T. Sougrati, F. Jaouen, B. Halevi, S. McKinney, G. McCool, C. Ma, X. Yuan, Z.-F. Ma, S. Mukerjee and Q. Jia, *Energy Environ. Sci.*, 2016, **9**, 2418-2432.
6. J. Tian, A. Morozan, M. T. Sougrati, M. Lefevre, R. Chenitz, J.-P. Dodelet, D. Jones and F. Jaouen, *Angew. Chem.-Int. Edit.*, 2013, **52**, 6867-6870.
7. H. Peng, Z. Mo, S. Liao, H. Liang, L. Yang, F. Luo, H. Song, Y. Zhong and B. Zhang, *Sci Rep*, 2013, **3**, 1765.
8. H. Peng, S. Hou, D. Dang, B. Zhang, F. Liu, R. Zheng, F. Luo, H. Song, P. Huang and S. Liao, *Appl. Catal. B-Environ.*, 2014, **158-159**, 60-69.
9. Y. Nabae, Y. Kuang, M. Chokai, T. Ichihara, A. Isoda, T. Hayakawa and T. Aoki, *J. Mater. Chem. A*, 2014, **2**, 11561-11564.
10. Y. J. Sa, D.-J. Seo, J. Woo, J. T. Lim, J. Y. Cheon, S. Y. Yang, J. M. Lee, D. Kang, T. J. Shin, H. S. Shin, H.

Y. Jeong, C. S. Kim, M. G. Kim, T.-Y. Kim and S. H. Joo, *J. Am. Chem. Soc.*, 2016, **138**, 15046-15056.
11. S. Yuan, J.-L. Shui, L. Grabstanowicz, C. Chen, S. Commet, B. Reprogle, T. Xu, L. Yu and D.-J. Liu, *Angew. Chem.-Int. Edit.*, 2013, **52**, 8349-8353.
12. G. Wu, M. Nelson, S. Ma, H. Meng, G. Cui and P. K. Shen, *Carbon*, 2011, **49**, 3972-3982.
13. C. Zhang, Y.-C. Wang, B. An, R. Huang, C. Wang, Z. Zhou and W. Lin, *Adv. Mater.*, 2017, **29**.
14. Q. Liu, X. Liu, L. Zheng and J. Shui, *Angew. Chem.-Int. Edit.*, 2018, **130**, 1218-1222.
15. Y. C. Wang, Y. J. Lai, L. Song, Z. Y. Zhou, J. G. Liu, Q. Wang, X. D. Yang, C. Chen, W. Shi, Y. P. Zheng, M. Rauf and S. G. Sun, *Angew. Chem.-Int. Edit.*, 2015, **54**, 9907-9910.
16. L. Yang, D. Cheng, H. Xu, X. Zeng, X. Wan, J. Shui, Z. Xiang and D. Cao, *Proc. Natl. Acad. Sci. U. S. A.*, 2018, DOI: 10.1073/pnas.1800771115.
